# Hypoxia-induced increases in glucose uptake do not cause oxidative injury or advanced glycation end-product (AGE) formation in vascular endothelial cells

**DOI:** 10.14814/phy2.12460

**Published:** 2015-07-15

**Authors:** Ryan J Viator, Heba Khader, Neha Hingorani, Sara Long, Victor Solodushko, Brian Fouty

**Affiliations:** 1 The Center for Lung Biology, University of South Alabama School of MedicineMobile, Alabama, USA; 2 The Department of Pharmacology, University of South Alabama School of MedicineMobile, Alabama, USA; 3 Department of Pharmacology, Zarqa UniversityZarqa, Jordan; 4 The Department of Internal Medicine/Division of Pulmonary and Critical Care Medicine, University of South Alabama School of MedicineMobile, Alabama, USA

**Keywords:** Advanced glycation endproducts, diabetes, endothelial cells, glucose, glucose transporter, pulmonary circulation

## Abstract

An increase in glucose uptake by endothelial cells exposed to hyperglycemia is the presumed initiating event that causes systemic vascular disease in individuals with diabetes. Diabetics do not develop clinically significant pulmonary vascular disease, however, despite the pulmonary circulation’s exposure to the same level of glucose. We hypothesized that pulmonary artery endothelial cells are protected from the detrimental effects of hyperglycemia because they take up less glucose than endothelial cells in the systemic circulation, either because of intrinsic differences between the two cell types or because the lower oxygen tension in the pulmonary arterial blood depresses glucose uptake. To test this hypothesis, we exposed normoglycemic and hyperglycemic bovine pulmonary artery (PAECs) and aortic endothelial cells (AECs) from the same animal to progressively lower oxygen tensions and determined glucose uptake. In contrast with our initial hypothesis, we detected no significant difference in glucose uptake between the two cell types. Furthermore, glucose uptake in both PAECs and AECs increased, not decreased, as the oxygen tension dropped; this oxygen-dependent increase in glucose uptake in endothelial cells predominated over the hyperglycemia-mediated decrease in glucose uptake that has been reported by others. Despite the increase in glucose uptake at lower oxygen tensions, we detected no corresponding increase in protein carbonylation or advanced glycation endproducts. These results demonstrate that small physiologically relevant changes in oxygen tension can have an important impact on glucose uptake in endothelial cells. These results also demonstrate that an increase in glucose uptake, by itself, is not sufficient to generate ROS-mediated protein carbonylation or increase intracellular advanced glycation endproducts in vascular endothelial cells.

## Introduction

Diabetes is a systemic disease in which the pancreas is unable to produce any (Type I) or sufficient (Type II) levels of insulin (Unger 1991; Virtanen and Knip 2003; Daneman 2006; Das and Rao 2007). Patients subsequently develop hyperglycemia, which is thought to be the initiating cause of the vascular complications that account for approximately 60% of all deaths in this disease (Seidell 2000; Aronson and Rayfield 2002; Lee et al. 2007; Action to Control Cardiovascular Risk in Diabetes Study G et al., 2008). Consistent with these clinical observations, genetic (db/db mice) (Kobayashi et al. 2000) and chemical (streptozotocin) (Matsumoto et al. 2007; Lopez-Lopez et al. 2008) animal models of diabetes demonstrate impaired acetylcholine-induced endothelium-dependent relaxation, an important pathophysiologic event indicative of endothelial cell dysfunction.

Hyperglycemia increases reactive oxygen species (ROS) production in aortic endothelial cells (AECs) which can cause cellular damage (Nishikawa et al. 2000; Brownlee 2001, 2005, 2007). Nishikawa et al. (2000) demonstrated that bovine AECs incubated in 30 mmol/L glucose produced threefold more mitochondrial-derived ROS than at baseline (5 mmol/L). This increase in mitochondrial ROS caused three potentially detrimental intracellular events: activation of the diacyl glycerol/protein kinase C pathway, the generation of advanced glycation end-products (AGE), and the accumulation of sorbitol due to activation of aldolase reductase. Activation of these pathways occurred, in part, by a ROS-mediated decrease in glyceraldehyde 3-phosphate dehydrogenase (GAPDH) activity (Du et al. 2003; Brownlee 2005). It has been postulated that it is the increase in glucose uptake by endothelial cells exposed to hyperglycemia that triggers the increase in ROS which ultimately leads to the endothelial dysfunction in diabetes (Du et al. 2000; Brownlee 2005; Cohen et al. 2006, 2007; Aronson 2008).

In contrast with the severe systemic vascular disease associated with diabetes, clinically relevant pulmonary vascular disease is uncommon in diabetics. While histologic changes can be seen in the pulmonary circulation of diabetic animals (thickening of the fused endothelial and epithelial basal laminae with an increased abundance of Weibel-Palade bodies in the pulmonary venules of streptozotocin-treated diabetic hamsters) (Popov and Simionescu 1997) and in autopsy studies in humans (thickened alveolar epithelial and pulmonary capillary basal laminae) (Weynand et al. 1999), the physiologic consequences of diabetes on the pulmonary vascular bed in humans appear limited to a slight (and inconsistent) reduction in the diffusing capacity of carbon monoxide (D_LCO_) (Boulbou et al. 2003; Ozsahin et al. 2006). In addition, while impaired acetylcholine-dependent relaxation has been demonstrated in pulmonary artery rings from streptozotocin-treated rats (although without detectable increases in pulmonary artery pressure) (Lopez-Lopez et al. 2008), diabetes is not a known risk factor for the development of pulmonary arterial hypertension in humans. Since both the pulmonary and systemic circulations are exposed to the same glucose concentrations, other factors must explain this difference in disease severity between the two vascular beds.

Endothelial cells in the pulmonary arteries are exposed to lower oxygen tension than endothelial cells in the systemic arteries (5% or 40 mmHg vs. 13% or 100 mmHg). Because it is the increase in glucose uptake by endothelial cells exposed to hyperglycemia that is the presumed reason for endothelial dysfunction in the systemic circulation (Kaiser et al. 1993; Du et al. 2000; Brownlee 2005; Cohen et al. 2006, 2007), we hypothesized that pulmonary artery endothelial cells (PAECs) take up less glucose than systemic endothelial cells due either to intrinsic differences between the two cell types or due to the lower oxygen tension in the pulmonary circulation. If true, this would protect the PAECs from the downstream activation of these deleterious pathways. To test this hypothesis, we studied bovine endothelial cells from both the systemic and pulmonary circulations to determine the effect of decreasing oxygen tension on glucose uptake and metabolism. Decreasing oxygen tension, even within the narrow physiologically relevant range that distinguishes the systemic (13%) from the pulmonary (5%) circulation, led to an increase, not a decrease, in glucose uptake; this occurred principally due to an upregulation of the insulin-independent glucose transporter 1 (GLUT-1). This increase in glucose uptake did not lead to an associated increase in AGE production or ROS-mediated protein carbonylation.

## Research Design and Methods

### Cell culture

Bovine AECs and PAECs from the same animal were purchased from Cell Applications (San Diego, CA) and maintained in PrimaPure™ Bovine Endothelial Cell Growth Medium (BECGM) (Genlantis, San Diego, CA). Cells were incubated in a humidified controlled gas flow incubator (Forma Series II water jacketed CO_2_ incubator, Thermo Electron Corporation, Marietta, OH) containing 5% CO_2_ at 37°C. Cells were grown to confluency, used between passages 6–15, and passaged using trypsin-EDTA. Confluent AECs and PAECs were incubated in a modular incubator chamber (Billups-Rothenberg, Del Mar, CA) containing 5% CO_2_ and one of the following: 0%, 5%, or 13% oxygen balanced with nitrogen at 37°C. Cells for 21% oxygen treatment were incubated in a humidified controlled gas flow incubator (Forma Series II water jacketed CO_2_ incubator; Thermo Electron Corporation, Marietta, OH) containing 5% CO_2_ at 37°C for the time period indicated in the respective figure legends.

### 3-O-Methyl-D-[1-^3^H]-glucose uptake assay

Glucose uptake was determined via the glucose uptake assay as previously described (Murray 1971) Briefly, cells were exposed to DMSO (vehicle) or 50 μmol/L cytochalasin B in DMEM (both without glucose) and 5 μCi 3-O-Methyl-D-[^3^H]-Glucose for 60–90 sec. Cells were rapidly washed five times with ice-cold glucose assay wash buffer (100 mmol/L MgCl_2_, 0.1 mmol/L Phloretin; Sigma-Aldrich, St. Louis, MO). Next, cells were lysed with 1 mL lysis buffer (200 mmol/L Na Acetate, 10% DMSO, 1% SDS; Sigma Aldrich, St. Louis, MO) and placed on ice until needed. Samples were transferred to disposable glass scintillation vials containing 6 mL Ready Safe™ liquid scintillation cocktail for aqueous samples (Beckman Coulter, Fullerton, CA). Radioactivity was measured by scintillation spectrometry on the LS 6500 Multi-Purpose Scintillation Counter (Beckman Coulter) for 1 min per sample. Protein concentration was determined by DC protein assay (Bio-Rad Laboratories, Hercules, CA) as per manufacturer’s instructions.

### Glucose depletion and lactate production

Glucose and lactate concentrations in the spent media were measured with the YSI 2300 STAT Plus Glucose-Lactate Analyzer version 2.25D (Global Medical Instrumentation, Inc., Ramsey, MN). The data are presented as mmol/L.

### SDS-PAGE and immunoblot analysis

Cells were washed, lysed in NP-40 lysis buffer (50 mmol/L Tris-HCl, 150 mmol/L NaCl, 1% NP-40, pH 8.0; Sigma Aldrich, St. Louis, MO) with protease and phosphatase inhibitors (P2714-1BTL, P5726-1ML, P2850-1ML; Sigma-Aldrich) and placed on ice. Protein concentration was determined by DC protein assay (Bio-Rad Laboratories, Hercules, CA). Protein was diluted in 2X SDS-PAGE sample buffer (62 mmol/L Tris-HCl, pH 6.8, 2% SDS, 10% glycerol, 0.001% bromophenol blue, 2% 2-mercaptoethanol, Sigma-Aldrich). Protein prepared for GLUT-1 detection was incubated at room temperature for a minimum of 15 min in 1:1 2X SDS-PAGE sample buffer before loading into wells. Proteins were resolved in 10% Tris-HCl polyacrylamide gel (Bio-Rad, Hercules, CA). and detected using rabbit anti-GLUT-1 (Chemicon, Temecula, CA) or anti-β-actin-HRP (Sigma, St. Louis, MO), followed by a goat anti-rabbit IgG-HRP (GE Healthcare, Little Chalfont Buckinghamshire, UK). Membranes were developed using Pierce Substrates (Rockford, IL).

### Determination of intracellular advanced glycation end products

Advanced Glycation End Product concentrations were determined using the OxiSelect™ Advanced Glycation End Product (AGE) ELISA kit (Cell Biolabs, Inc. San Diego, CA) following manufacturer’s instruction. AGEs were detected using an anti-AGE antibody with a HRP-conjugated secondary antibody and detected spectrophotometrically at 450 nm. Absorbances were measured by the SpectraMax M5 Multi-Functional Plate Reader (Molecular Devices, Sunnyvale, CA). Data are presented as μg/mL.

### Reactive oxygen species production

Reactive oxygen species production was measured by the OxiSelect™ Protein Carbonyl ELISA kit (Cell Biolabs, Inc.), an indirect measurement of ROS production. This assay involves derivitization of the carbonyl group with dinitrophenylhydrazine (DNPH), followed by detection with a anti-DNP antibody detection, which can be measured spectrophotometrically at 375 nm (Reznick and Packer 1994; Talent et al. 1998). Oxidized BSA served as the standard control. Confluent samples were incubated in the respective oxygen tensions for 4 days. Cells were then sonicated in lysis buffer (25 mmol/L HEPES, 150 mmol/L NaCl, 10 mmol/L MgCl_2_, 1 mmol/L EDTA, 2% glycerol, pH 7.5), and protein concentration was determined by BCA protein assay (Pierce Protein Research Products, Rockford, IL). Absorbances were measured by the SpectraMax M5 Multi-Functional Plate Reader (Molecular Devices).

### Statistical analyses

Data are expressed as means ± standard error of the mean (SEM) of at least three independent experiments measured in triplicate. Data were analyzed using ANOVA combined with Tukey’s Multiple Comparison Test post-hoc analysis, with a *P* value <0.05 considered significant.

## Results

### Vascular endothelial cells take up more glucose as oxygen tension decreases

We examined the effect of decreasing oxygen tension on glucose uptake in vascular endothelial cells. Bovine pulmonary artery and AECs from the same animal were grown to confluence in 21% oxygen and 5.5 mmol/L glucose. Cells were then placed in fresh media and exposed to 21%, 13%, 5%, and 0% oxygen for 2 days to allow cells to acclimate. Media was then replaced with fresh media (pre-incubated at the same oxygen tension to prevent the effects of re-oxygenation) and glucose uptake over the next 48 h was determined. To calculate the amount of glucose taken up by cells, the media was sampled and the glucose concentration was subtracted from the initial concentration of glucose.

**Figure 1:**
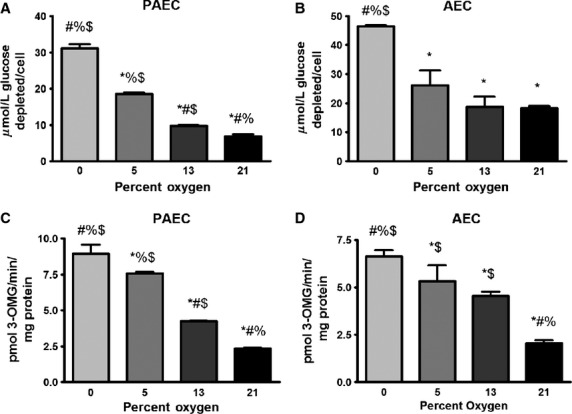
Aortic endothelial cells (AECs) and pulmonary artery endothelial cells (PAECs) take up more glucose as oxygen tension decreases. (A) and (B): Confluent endothelial cells in 5 mmol/L glucose were exposed to different oxygen tensions for 2 days. Media was then replaced with fresh media (pre-incubated at the specified oxygen tension to prevent the effects of re-oxygenation). Forty-eight hours later, spent media was collected and glucose concentration in the media was determined for bovine PAECs (A) and AECs (B). Glucose depletion was normalized to cell number. (C) and (D): Confluent cells were exposed to different oxygen tensions for 4 days in 5 mmol/L glucose and then radiolabeled glucose uptake determined using 5 μCi 3-OMG in the presence of 5 mmol/L cold D-glucose in bovine PAECs (C) and AECs (D). Radiolabeled glucose uptake was normalized to protein concentrations. Data represent three independent experiments each done in triplicate. *P < 0.05 compared to 0% oxygen; ^#^*P* < 0.05 compared to 5% oxygen; ^%^*P* < 0.05 compared to 13% oxygen; ^$^*P* < 0.05 compared to 21% oxygen.

To first determine the effectiveness of the hypoxia chambers, the partial pressure of oxygen of the media was determined after 48 h and were as follows: 21% oxygen (room air): 146 mmHg; 13% oxygen: 104 mmHg 5% oxygen: 45 mmHg; 0% oxygen: 20 mmHg. We were unable to achieve complete anoxia (0 mmHg) even after prolonged exposure to 0% gas. As the oxygen tension decreased, glucose depletion from the medium (equivalent to glucose uptake) increased in both pulmonary artery and AECs (Fig.1A and B). To confirm these findings with an alternate method of assessing glucose uptake, we measured radiolabeled glucose uptake in each cell type after 4 days of similar treatment. Confluent PAECs and AECs were exposed to 21%, 13%, 5%, and 0% oxygen for 4 days in 5 mmol/L glucose and then glucose uptake was determined using 3-O-Methyl-D-[1-^3^H]-Glucose (3OMG). Similar to the findings obtained from the glucose depletion assay, glucose uptake increased as the oxygen tension decreased, an effect that was seen in both pulmonary artery and AECs (Fig.1C and D). (While there was some differences in glucose uptake between PAECs and AECs depending on the method used, these differences were not statistically significant and did not indicate to us an intrinsic difference in glucose uptake between the cell types. In contrast, the effect of lowering oxygen tension was consistent in both cell types regardless of the method used.) Incubating cells with the glucose transporter 1 (GLUT-1) inhibitor, cytochalasin B, blocked greater than 80% of glucose uptake (data not shown) consistent with previous observations that in endothelial cells most of the glucose enters through GLUT-1 (Mann et al. 2003).

### GLUT-1 protein expression increases as oxygen tension decreases

**Figure 2:**
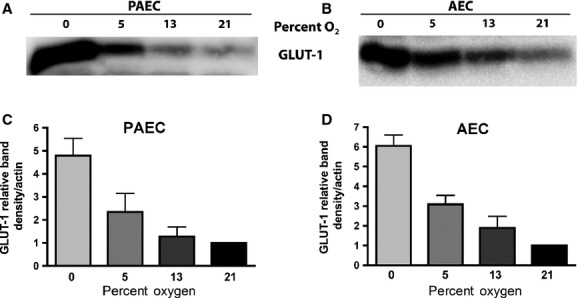
Bovine pulmonary artery and aortic endothelial cells upregulate GLUT-1 protein as oxygen tension decreases. Confluent cells were exposed to various oxygen tensions for 4 days. Cells were then lysed and 5 µg of cell lysates resolved by SDS-PAGE. Representative blot (A and B) and densitometry from four independent experiments (C and D).

The previous data demonstrated an increase in glucose uptake with decreasing oxygen tension, an increase that appeared to be due mainly to augmented glucose transport through GLUT-1. We next examined the effect of decreasing oxygen tension on GLUT-1 protein expression. PAECs and AECs were grown to confluence in 21% oxygen and 5 mmol/L glucose. Cells were then placed in fresh media under different oxygen tensions and cell lysates harvested after 48 h. GLUT-1 protein expression was determined using Western Blotting. GLUT-1 expression increased as oxygen tension decreased (Fig.2). The increase was most dramatic at 0% oxygen, but could be detected at both 5% and 13% oxygen.

### Increased lactate formation as oxygen tension decreases

**Figure 3:**
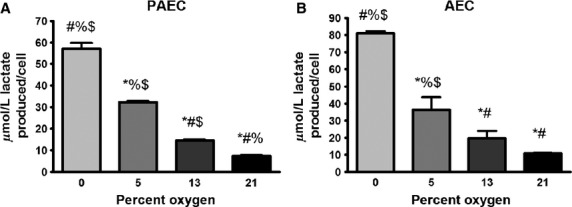
Aortic endothelial cells (AEC) and pulmonary artery endothelial cells (PAECs) increase lactate production as oxygen tension decreases. Confluent endothelial cells in 5 mmol/L glucose were exposed to different oxygen tensions for 2 days. Media was then replaced with fresh media (pre-incubated at the specified oxygen tension to prevent the effects of re-oxygenation). Forty-eight hours later, spent media was collected and lactate concentration in the media was determined for bovine PAECs (A) and AECs (B). Lactate concentration was normalized to cell number. Data represent three independent experiments, each done in triplicate. *P < 0.05 compared to 0% oxygen; ^#^*P* < 0.05 compared to 5% oxygen; ^%^*P* < 0.05 compared to 13% oxygen; ^$^*P* < 0.05 compared to 21% oxygen.

To determine the fate of glucose taken up by cells under the different oxygen tensions, cells were grown to confluence in 21% oxygen and 5 mmol glucose, provided with fresh media, exposed to the four different oxygen tensions and then lactate levels in the media determined after 4 days. Lactate production was calculated by subtracting the initial level from the level present at day 4. Lactate production normalized to cell number increased as oxygen tension decreased from 21% to 0% (Fig.3). The increase in lactate from baseline (21%) was detectable even at 13% and 5% oxygen, oxygen tensions not normally considered low enough to increase HIF-1α▫(Jiang et al. 1996) or stimulate anaerobic metabolism. The percent of glucose converted to lactate ranged from 30% in 21% oxygen to approximately 90% in 0% oxygen (data not shown).

### Decreasing oxygen tension opposes the hyperglycemia-induced downregulation of glucose uptake in endothelial cells

**Figure 4:**
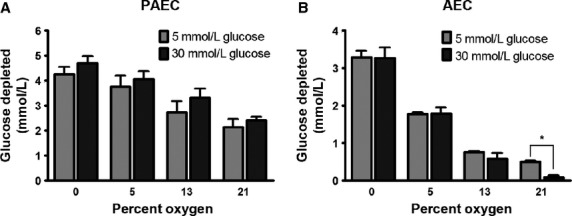
Hypoxia-induced increases in glucose uptake predominate over hyperglycemia-induced decreases in glucose uptake in bovine AECs. Cells were grown to confluence in 21% oxygen and 5 mmol/L glucose. Cells were then incubated in either 5 or 30 mmol/L glucose at 21%, 13%, 5%, or 0% oxygen for 48 h. Media was then replaced by fresh media of the same glucose concentration and oxygen tension for an additional 24 h. Spent media was then collected and glucose concentration in the media was determined for bovine PAECs (A) and AECs (B). Glucose concentration was normalized to cell number. Data represent three independent experiments, each done in triplicate.

Alpert and colleagues have demonstrated that bovine AECs exposed to high glucose (30 mmol/L) decreased glucose uptake by downregulating GLUT-1 protein expression (Alpert et al. 2002, 2005). The data presented above indicates that as oxygen tension decreased, the amount of glucose taken up by PAECs and AECs increased. To determine the relative strengths of each stimulus, cells were exposed to both conditions simultaneously and the effect on glucose uptake assessed. PAECs and AECs were grown to confluence in 21% oxygen and 5 mmol glucose. Cells were then incubated in either 5 or 30 mmol glucose at 21%, 13%, 5%, or 0% oxygen for 48 h. Fresh media of similar glucose concentration (and pre-incubated at the proper oxygen tension) was then added and glucose depletion from the media was determined for the next 24 h. Consistent with the results of Alpert et al. (2002, 2005), glucose uptake in AECs exposed to both 21% oxygen and 30 mmol glucose was reduced compared to AECs exposed to 21% oxygen and 5 mmol glucose (Fig.4B). The decrease in glucose uptake in PAECs exposed to 30 mmol glucose and 21% oxygen did not reach statistical significance (Fig.4A). As the oxygen tension was decreased, however, glucose uptake increased in both cell types, even in the AECs exposed to 30 mmol/L glucose. The inhibitory effect of hyperglycemia on glucose uptake in the AECs was completely reversed when the oxygen tension was decreased to 13% oxygen (Fig.4).

### No increase in intracellular protein carbonylation despite increase in glucose uptake

**Figure 5:**
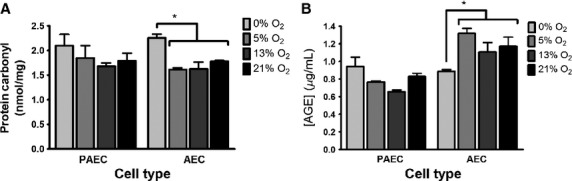
Neither protein carbonyl nor AGE concentrations were increased in bovine vascular endothelial cells as oxygen tension decreased. Confluent cells were incubated for 4 days in different oxygen tensions. Protein carbonyl and AGEs were determined using ELISA kits and normalized to mg of protein. Data represent three independent experiments each done in triplicate. *P < 0.05.

Increased glucose uptake is the presumed mechanism through which hyperglycemia induces vascular endothelial cell dysfunction. Some of this endothelial injury is believed to be due to the generation of ROS and AGEs within the cell (Giardino et al. 1996; Nishikawa et al. 2000; Brownlee 2001; Aronson and Rayfield 2002; Du et al. 2003; Aronson 2008). Proteins can be modified by ROS causing protein carbonylation which leads to irreversible structural changes that can alter protein functions (Kim et al. 2008). Changes in protein carbonylation and AGEs were measured using commercially available ELISA kits in bovine endothelial cells exposed to 0%, 5%, 13%, and 21% oxygen for 4 days. Despite the increased glucose uptake associated with a decrease in oxygen tension, there was no corresponding increase in protein carbonylation in either AECs or PAECs (Fig.5A). Similarly, AGE levels did not increase in either PAECs or AECs as oxygen tension decreased (Fig.5B). These data suggest that the increased glucose uptake that occurred as oxygen tension decreased did not lead to a corresponding increase in ROS-mediated protein carbonylation or AGEs.

## Discussion

The endothelial cells of over 25 million Americans are chronically exposed to elevated levels of glucose (Aronson and Rayfield 2002; Action to Control Cardiovascular Risk in Diabetes Study G et al., 2008). This includes not only individuals with Type I and Type II diabetes, but also critically ill patients with stress-induced hyperglycemia (van den Berghe et al. 2001; Van den Berghe 2005). Yet the complications of this hyperglycemia are borne primarily by systemic endothelial cells (Aronson and Rayfield 2002; Lee et al. 2007; Aronson 2008; Brouwers et al. 2010), mostly sparing the pulmonary circulation. The currently accepted paradigm in diabetes, is that it is an *increased* glucose uptake in endothelial cells exposed to hyperglycemia that leads to the vascular dysfunction characteristic of that disease. Therefore, to explain the different effects of diabetes on the two circulations, we speculated that when exposed to hyperglycemia endothelial cells in the pulmonary circulation might take up less glucose than endothelial cells in the systemic circulation, either because of intrinsic differences between the cell types or, alternatively, because the lower oxygen environment in the pulmonary circulation would depress glucose uptake. We tested this hypothesis using paired AECs and PAECs from the same animal exposed to different, physiologically relevant, oxygen tensions.

In this manuscript, we demonstrate that our initial hypothesis was incorrect. First, there was no significant or consistent difference in glucose uptake between AECs and PAECs across the various oxygen tensions. Second, we demonstrated that in both PAECs and AECs, glucose uptake increased, not decreased, as oxygen tension dropped, an effect that was due primarily to an upregulation of the facultative glucose transporter GLUT-1. While this observation seems intuitive given that GLUT-1 is a HIF-1α responsive gene, this increase in glucose uptake and protein expression occurred even at the relatively modest reductions in oxygen tension that distinguish the systemic (13% oxygen) from the pulmonary (5% oxygen) circulation; in contrast, others have demonstrated that HIF-1α upregulation was not detected in cell culture until oxygen levels decreased to about 1.5–2.0% (Jiang et al. 1996).

We also demonstrate herein that the hyperglycemia-mediated *decrease* in glucose uptake in AECs, which has been reported by others (Alpert et al. 2002, 2005; Totary-Jain et al. 2005; Riahi et al. 2010), is abolished as the oxygen tension decreases. Whether hyperglycemia increases or decreases glucose uptake in endothelial cells is an area of disagreement in the literature. A paper by Kaiser et al. (1993) demonstrated that vascular (aortic) endothelial cells exposed to hyperglycemia did not decrease radiolabeled glucose uptake whereas vascular (aortic) smooth muscle cells did. The endothelial cell’s failure to downregulate glucose uptake was postulated to be a critical factor in mediating the deleterious effect of hyperglycemia on endothelial cells. This paper has been frequently cited to explain the potential toxic effects of hyperglycemia on endothelial cells (Hahn et al. 1998; Meier and King 2000; Busik et al. 2002; Creager et al. 2003; Giacco and Brownlee 2010; Shaw et al. 2014). Subsequent papers by the same lab, however, demonstrated that AECs exposed to hyperglycemia did, in fact, decrease glucose uptake (and decrease GLUT-1 expression), but it required 36–48 h to occur (Alpert et al. 2002, 2005; Totary-Jain et al. 2005; Cohen et al. 2006; Riahi et al. 2010) rather than the 24 h that was the timepoint examined in their original paper (Kaiser et al. 1993). This downregulation was due, at least in part, to an increased production of 12-hydroxyeicosatetraenoic acid and could be blocked by inhibiting the enzyme *12-lipoxygenase* with esculetin (Alpert et al. 2002, 2005). Additional work by these same investigators demonstrated that calreticulin, which is upregulated in vascular smooth muscle and endothelial cells exposed to hyperglycemia, binds to *Cis*-acting elements in the 3′ untranslated region of GLUT-1, rendering GLUT-1 mRNA susceptible to degradation (Totary-Jain et al. 2005).

Those studies (Kaiser et al. 1993; Alpert et al. 2002, 2005; Du et al. 2003; Totary-Jain et al. 2005; Riahi et al. 2010) were all performed in 21% oxygen, however. Unless a patient is on supplemental oxygen, vascular endothelial cells are never exposed to 21% oxygen, a tension that generates a partial pressure of oxygen (PO_2_) of 140–160 mmHg. In the experiments included in this manuscript, we carefully controlled the oxygen tension to determine whether small, but physiologically relevant changes altered the amount of glucose taken up by endothelial cells. Using 21% oxygen allowed us to compare our results to the bulk of published studies in this area, most of which use this oxygen tension (Nishikawa et al. 2000; Alpert et al. 2002, 2005; Du et al. 2003; Cohen et al. 2006); 13% was studied since it represents the oxygen tension seen by most systemic arterial endothelial cells in vivo; 5% was studied since it represents the oxygen tension experienced by PAECs (and systemic veins) in vivo; 0% was used to simulate complete hypoxia (even though we were unable to achieve complete anoxia (0 mmHg). As the percent of oxygen decreased, endothelial cells took up more glucose, an increase that was seen in both normoglycemic and hyperglycemic conditions. Perhaps most importantly, the inhibitory effect of hyperglycemia on glucose uptake in AECs was completely eliminated once the oxygen tension was decreased to 13% (i.e. normal for AECs in vivo).

Despite the increased glucose uptake associated with the decrease in oxygen tension, however, neither PAECs nor AECs demonstrated an increase in intracellular AGE formation or ROS-mediated protein carbonylation in our study. Nishikawa et al. (2000) demonstrated that bovine AECs incubated in 30 mmol/L glucose produced threefold more mitochondrial-derived ROS than at baseline glucose (5 mmol/L). This increase in mitochondrial ROS caused three potentially detrimental intracellular events: activation of the diacyl glycerol/protein kinase C pathway, the generation of advanced glycation end-products, and the accumulation of sorbitol due to activation of aldolase reductase. While Nishikawa and colleagues demonstrated that hyperglycemia led to activation of these ROS-mediated pathways (Nishikawa et al. 2000), experiments to confirm that hyperglycemia actually increased glucose uptake by the endothelial cells were not performed. Based on the studies cited herein, hyperglycemia more likely led to a *decrease*, not an increase, in glucose uptake in those AECs since the experiments were performed in 21% oxygen.

If these in vitro observations are true in vivo, endothelial cells in the pulmonary arterial circulation would be expected to take up more, not less, glucose than systemic arterial endothelial cells in both normoglycemia and hyperglycemia. How this possibility can be reconciled with the hypothesis that it is an increase in glucose uptake that is toxic to endothelial cells in diabetes will require further investigation. There may be other reasons the pulmonary circulation is relatively protected in diabetes: the increased glucose uptake seen with lower oxygen tensions may inhibit mitochondrial respiration (the Crabtree effect) (Mustea and Bara 1979; Krutzfeldt et al. 1990) thus potentially decreasing ROS production from the mitochondria; the lower pressure in the pulmonary circulation may alter glucose uptake and metabolism rendering the increase in glucose uptake less injurious; there may be intrinsic differences in glucose uptake and metabolism between PAECs and AECs that we were unable to detect in our experiments, particularly since cell exposure to hyperglycemia here was significantly shorter than the long-term exposure endothelial cells experience in vivo. At a minimum, however, these studies indicate that an increase in glucose uptake, by itself, is not sufficient to cause increased AGE formation or ROS-mediated protein carbonylation and suggest that the currently accepted mechanism through which hyperglycemia inflicts injury upon endothelial cells is incomplete.

## Conflict of Interest

None declared.
